# A Simple, Ecofriendly, and Fast Method for Nitrate Quantification in Bottled Water Using Visible Spectrophotometry

**DOI:** 10.3390/toxics12060383

**Published:** 2024-05-23

**Authors:** Wellington Diego da Ascenção, Caroline Cristine Augusto, Vitor Hugo Soares de Melo, Bruno Lemos Batista

**Affiliations:** 1 Federal University of ABC (UFABC), Dean of Undergraduate Studies, 5001 Avenida dos Estados Avenue, Santo André 09210580, SP, Brazil; wellington.ascencao@ufabc.edu.br (W.D.d.A.); vitor.melo@ufabc.edu.br (V.H.S.d.M.); 2 Center for Natural and Human Sciences, Federal University of ABC (CCNH/UFABC), 5001 Avenida dos Estados Avenue, Santo André 09210580, SP, Brazil; caroline.augusto@ufabc.edu.br

**Keywords:** cadmium free, carcinogenic, Griess reaction, zinc

## Abstract

There are many works associating the presence of nitrate in water and the occurrence of cancer in humans. The most common method for quantifying nitrate in water is based on the use of toxic cadmium as a reductant. In this work, a new approach was developed for the quantification of nitrate in bottled water with indirect spectrophotometry using Zn^0^ as a reductant. Nitrate is reduced to nitrite using Zn^0^ in a buffered medium (acetate/acetic acid) and quantified with visible spectrophotometry using the Griess reaction between sulfanilamide and N-(1-naphthyl)-ethylenediamine. The influence of pH, buffer solution (constitution and concentration), Zn^0^ (mass and granulometry), and agitation time on the efficiency of nitrite generation was evaluated. The optimal conditions were an acetate–acetic acid buffer solution with a concentration and pH of 0.75 mol L^−1^ and 6.00, respectively, and a Zn^0^ particle size of 20 MESH and Zn^0^ mass of 300 mg. The limits of detection and quantification (LoD and LoQ) were 0.024 and 0.08 mg L^−1^, respectively. The method’s accuracy and precision were evaluated using the analysis of commercial bottled water. In conclusion, the use of Zn^0^ instead of cadmium provided a green method with excellent LoD/LoQ. Further, the method proved to be simple and easy to apply during outdoor analysis.

## 1. Introduction

Nitrate is the most commonly found form of nitrogen and is naturally present in soils, waters, plants and meat, making it of great environmental interest [1,2]. The precise and accurate quantification of the nitrate in water for human consumption has become an object of interest in public health due to the association between nitrate intake through water and the incidence of methemoglobinemia and cancer [1,3,4,5]. Nitrate consumption is associated with the potential formation of nitrosamines and nitrosamides, which are carcinogens for humans [6]. In the USA and Brazil, the limit of nitrate (in mg of N per liter) in drinking water is 10 mg L^−1^ [7,8]. According to Brazilian regulations, the maximum allowed value for nitrate in bottled water is 50 mg L^−1^ [9].

Despite several analytical methods available, the quantification of nitrate in aqueous solutions is not a trivial task. Most of the analytical methods describe complex procedures associated with limited ranges of concentration and interferents [10]. There are many methods in the literature for quantifying nitrate in water based on the most diverse analytical techniques: chromatography, electrophoresis, chemiluminescence, electrochemistry (potentiometry and voltammetry), electrochemiluminescence, and, the most important for this work, spectrophotometry [11,12,13].

Nitrate can be directly quantified using several separation techniques, such as high-performance liquid chromatography (HPLC), ion chromatography (IC), and capillary electrophoresis (CE), techniques that are relatively expensive [7,12]. IC is one of the most used chromatographic methods for quantifying nitrate in water because it allows for the simultaneous determination of nitrate with other anions of analytical interest, such as phosphate, sulfate, nitrite, and chloride, among others [11,13]. The columns used in HPLC and IC are very susceptible to the sample’s impurities [13]. Therefore, these samples require filtration and other sample treatments to extend the useful life of these columns [11,12,13]. IC, the most common method for quantifying nitrate in aqueous matrices, has quantification limits in the order of 0.10 mg L^−1^ [13]. CE is also a very versatile separation technique that can be used to quantify cationic, anionic, and neutral substances [13]. CE has several advantages, such as high separation efficiency, short analysis time, and very low injection volumes (in the order of nL), and can be directly applied to various matrices. Its disadvantages include the restricted capacity of the capillary columns and the low sensitivity of the UV detector due to the small diameter and short optical path of the capillary [13]. 

Electrochemical techniques, especially voltammetry and potentiometry, can also be used for nitrate determination [13]. The low cost and portability of electroanalytical devices provide a number of attractive methods [12]. Potentiometric methods for nitrate quantification are very common [11,12,13]. The most common technique is the use of ion-selective electrodes (ISEs) [13]. Detection limits for nitrate are from 10^−5^ to 10^−2^ mol L^−1^ [13]. For voltammetry, platinum, gold, copper, diamond, glassy carbon, and transition metal oxide electrodes are used [12]. However, the application of electrodes is limited because their surface is easily poisoned by other species, thus reducing analytical sensitivity and precision [13]. Electrochemical techniques based on various modified electrodes are favored due to their simplicity, high sensitivity, and selectivity [12,13]. Various methods and analytical techniques have been presented in recent years to improve the surface of electrodes in order to obtain high specificity and catalytic activity for redox reactions [12]. In recent years, nanomaterials have attracted interest [14]. Nanoelectrodes can be applied very successfully in electrochemical detection. When applied to modify surfaces, they improve the electrocatalytic capacity of electrodes as reducing agents [14]. Nanoparticles, with their small size and large surface area, constitute excellent electrocatalysts [14]. In addition to nanoparticles, nanodots, nanotubes, nanoshells, nanoclusters, nanofibers, and nanocomposites have also been applied to electrochemical sensors [14]. The exceptional properties of nanoelectrodes offer low detection levels of μmol L^−1^ or even nmol L^−1^, as well as wide detection ranges [14].

**Figure 1:**
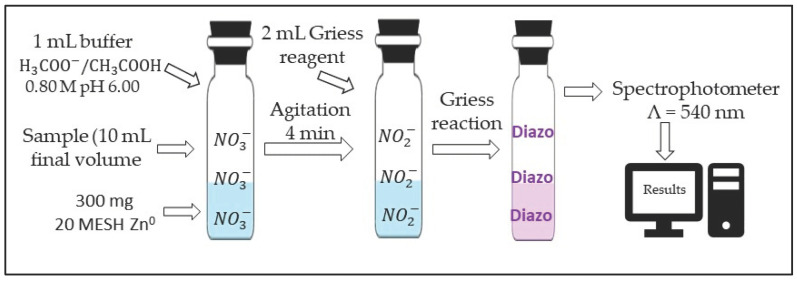
Sequential steps for the reactions using the proposed method.

Spectrophotometric techniques are, by far, the most used in the quantification of nitrate in aqueous matrices, as they have an excellent limit of quantification (~1 mg L^−1^ or less, as in the case of fluorimetric methods) and represent a simple methodology compared with the previously methods [13]. The most widespread spectrophotometric methods in the literature for quantifying nitrate in aqueous matrices use the reduction of nitrate to nitrite in a cadmium column, followed by color development by diazotation reaction, specifically the Griess reaction (Figure 1) [11,13]. The most important disadvantage is that cadmium is a highly toxic carcinogen to humans [15]. Some authors have developed new alternatives, such as replacing cadmium with zinc as a nitrate to a nitrite reducer in mixtures with barium and manganese salts to determine nitrate levels in plant tissues [14]. Other studies combined zinc in cadmium columns to improve nitrate reduction efficiency [15]; however, these methods did not overcome the presence of cadmium. 

Recently, some authors have used zinc only to reduce nitrate to nitrite, which is quantified by the sulfanilamide and N-(1-naphthyl)-ethylenediamine (NED) method [16,17,18]. Merino [16] used zinc metal (Zn^0^) powder in an ammonia-based buffer (pH = 11) for quantification. The use of metallic zinc powder requires filtration, as zinc powder interferes with spectrophotometric measurements. Furthermore, the ammonia-based buffer is volatile and harmful to the analyst. On the other hand, Murray et al. [17] used Zn^0^ with a grade of 150 µm and did not use the buffering of the medium to carry out the reaction to reduce nitrate to nitrite. Probably, the reaction efficiency was affected by the absence of a buffer solution, compromising the limits of detection (LoD) and quantification (LoQ). Ellis et al. [18] reduced nitrate to nitrite using Zn^0^ and a citrate/citric acid buffer in a flow injection analysis to quantify nitrate in estuarine waters. Although the method used presented good analytical parameters, its cost is high, and its operation is somewhat complex, which is rarely found in routine analysis laboratories.

Therefore, this work aimed to develop a safer and simpler spectrophotometry method for the quantification of nitrate in water samples by using the Griess reaction. The use of Zn^0^ instead of toxic cadmium was evaluated as a safer method for the environment and the analytical chemist. A systematic study was also carried out for method validation and tested on real samples.

## 2. Materials and Methods

### 2.1. Reagents and Equipment

Type 1 water was used in all experiments. Sodium hydroxide, sulfuric acid, hydrochloric acid, sodium acetate, acetic acid, anhydrous monobasic potassium phosphate, dibasic sodium phosphate, citrate tribasic sodium dihydrate, sodium bicarbonate, and sodium carbonate were of analytical grade (Vetec, Rio de Janeiro, Brazil). Citric acid and N-(1-naphthyl)-ethylenediamine were purchased from Sigma Aldrich (St. Louis, MO, USA). Potassium nitrate, sodium nitrite, sulfanilamide, and metallic zinc (granulometry of 20 MESH) were purchased from Neon (Suzano, SP, Brazil), Dynamic (Indaiatuba, SP, Brazil), Synth (São Paulo, Brazil), and Acros (Antwerp, Belgium), respectively. Colorimetric reagents, nitrate stock solutions, nitrite stock solutions, and buffer solutions were prepared weekly and kept under refrigeration (4 °C).

All spectrophotometric measurements were performed in the UV/VIS range using a spectrophotometer (Hach DR 5000, Loveland, CO, USA) and a glass cuvette with a 10 mm optical path. Analytical balances (Marte AY-220, and Marte BL320H, Valinhos, SP, Brazil) and pHmeter (Del Lab model DLA PH, Araraquara, SP, Brazil) were used for the experiments. For comparison between methods, an IC (Metrohm 883 Basic IC Plus, Herisau, Switzerland) was used in the following operational conditions: conductance detector, anion exchange column, pump flow of 1 mL min^−1^, mobile phase formed by ${CO}_{3}^{2-}$ 1.8 mmol L^−1^, ${HCO}_{3}^{-}$ 1.7 mmol L^−1^, and regenerating solution $H_{2}{SO}_{4}$ at 50 mmol L^−1^. For IC analysis, samples were filtered through a 0.45 μm syringe filter.

### 2.2. Solutions

Solutions of ${CO}_{3}^{2-}$ 1.8 mmol L^−1^, ${HCO}_{3}^{-}$ 1.7 mmol L^−1^, regenerating solution $H_{2}{SO}_{4}$ 50 mmol L^−1^, sodium hydroxide (10 mol L^−1^), and hydrochloric acid (6 mol L^−1^) were prepared daily. The colorimetric reagent (Griess reagent) was prepared by dissolving 2.5 g of sulfanilamide, 25 mL of phosphoric acid, and 0.25 g of N- (1-naphthyl)-ethylenediamine, and the volume was made up to 250 mL with water [11]. A nitrate stock solution (1000 mg L^−1^) was prepared by dissolving dried (105 °C for 24 h in a lab oven) potassium nitrate and 1 mL of CHCl_3_ in water. A nitrite stock solution (1000 mg L^−1^) was prepared by dissolving dried (105 °C for 1 h in a lab oven) sodium nitrite and 1 mL of CHCl_3_ in water. A 0.75 mol L^−1^ acetate–acetic acid buffer (pH = 6.10) was prepared by dissolving sodium acetate and glacial acetic acid in water, and the pH was set to 6.10 with sodium hydroxide at 10 mol L^−1^. Other acetate–acetic acid buffer solutions at different pHs were prepared daily for tests. We tested other buffer solutions (0.750 mol L^−1^ and pH = pK_a_ of conjugated acid) of bicarbonate–carbonate, monobasic phosphate–dibasic phosphate, and citric acid–citrate. The solutions were prepared daily.

### 2.3. Reduction Reaction of Nitrate to Nitrite and Diazotation Reaction

All samples were prepared and analyzed according to the scheme in Figure 1. The agitation consisted of the repeated vigorous movement of the tube in a vertical direction, upwards and downwards. Several parameters (pH, concentration, the nature of the buffer solution, zinc mass, and stirring time) that influence the reduction of nitrate to nitrite were studied following the procedure in Figure 1.

### 2.4. Preparation of Calibration Standards

The calibration standards for nitrate quantification (0.2 to 1.0 mg L^−1^ ${NO}_{3}^{-}$) were prepared daily according to the procedure in Figure 1. For nitrite quantification (0.15–0.74 mg L^−1^ ${NO}_{2}^{-}$), a method from the literature was used [11]. All measurements were performed in triplicate at a 540 nm wavelength. 

### 2.5. Sampling and Preparation

The samples (n = 20) of uncarbonated natural waters were from quality-recognized manufacturers purchased at local supermarkets. The pH and conductivity at 25 °C varied from 5.25 to 9.58 and 122 to 301 µS/cm, respectively. The concentrations (in mg/L) of NaHCO_3_, Ba^2+^, Na^+^, Ca^2+^, K^+^, Mg^2+^, Cl^−^, F^−^, Br^−^, Sr^2+^, ${PO}_{4}^{3-}$, and ${SO}_{4}^{2-}$ varied from 7 to 287, 0.02 to 0.41, 1.2 to 36, 1 to 31, 0.4 to 31.3, 0.3 to 16.5, 0.1 to 10.9, 0.02 to 0.34, 0.01 to 0.05, 0.01 to 0.35, 0.05 to 0.39, and 0.17 to 8.52, respectively. Nitrite was quantified according to the literature [11], and nitrate was quantified using the proposed method. The samples were treated according to the procedure in Figure 1, and all analyses were performed in triplicate for each manufacturer of bottled natural water. Samples were diluted (1 + 9 mL) in type 1 water. Blanks were run in each batch of analysis.

### 2.6. Analytical Parameters and Statistics

In the present work, the following figures of merit were determined: selectivity, working range, linearity, LoD, LoQ, trueness (expressed quantitatively in terms of bias), repeatability, and intermediate precision. The validation process complied with several national and international validation protocols [19,20,21,22,23]. Blanks were analyzed to study the effects of matrix and recovery tests. 

The selectivity and bias were evaluated by comparing the reference method (IC) with the present method [19,22,24]. For comparisons, the *F* test was applied to assess the homogeneity of variances. The Student’s *t* test was used when ($\bar{x_{1}}$ − $\bar{x_{2}}$) differed significantly from zero (confidence interval of 95%), where $\bar{x_{1}}$ and $\bar{x_{2}}$ are the concentrations of a sample analyzed using the two different methods [25]. In addition, the bias was determined with recovery tests using three concentrations of the analytical curve: low, medium, and high [19,22,24]. 

The matrix effect was evaluated by comparing the analytical curves of the standards prepared in water and standards prepared using bottled water. The *F* test was applied to assess the homogeneity of variances, and the Student’s *t* test was applied to compare the angular coefficients of both analytical curves (confidence interval of 95%) [22,26].

In the working range, after blanks, the first calibration point (0.2 mg L^−1^) of the analytical curve was chosen with an order of magnitude above the LoQ (0.08 mg L^−1^) and the other four values of the working range (0.4–1.0 mg L^−1^). Linearity was evaluated by applying the blank test to verify the homoscedasticity of the linear regressions. Also, the correlation coefficient (R^2^) and the residual graph of these regressions were determined [20]. 

The LoD was determined as three times the standard deviation of 10 blank measurements divided by the slope of the calibration curve. LoQ was calculated as 3.3 LoD [22,24].

For the calculation of the standard uncertainties, the uncertainty of the calibration curve, of the repeatability, and of the intermediate precision were considered. The uncertainty was calculated as the expanded uncertainty U, which was obtained by multiplying the combined standard uncertainty (μ_c_y) by a coverage factor k, which is equal to two in a confidence interval of 95% [19,22]. The results, x, of the nitrate concentrations, C_N_, in the samples and their respective uncertainties were reported as “x = C_N_ ± U mg L^−1^”, where C_N_ is the average of the nitrate concentrations obtained in triplicates of measurements [19,22]. 

The precision was evaluated by repeatability and intermediary precision [20,22]. Repeatability was determined on the same day and using the same reagents and equipment [20,22]. On the other hand, intermediate precision was determined in two ways: using the same reagents and equipment, albeit on different days, and using different reagents and equipment, but on the same days [20,22].

The repeatability was determined using the following expression:

$$RSD=\frac{s}{M}\times 100,$$

where *RSD* is the relative standard deviation in recovery tests at three concentration levels of the analytical curve, *s* is the standard deviation, and *M* is the mean of the measurements. 

The *F* test (with a confidence interval of 95%) was applied to assess the homogeneity of variances to determine the intermediate precision.

## 3. Results and Discussion

### 3.1. Nitrate and Nitrite Reduction Reactions

After agitation (Figure 1), the reduction reaction of nitrate to nitrite occurs. The reactions using Zn^0^ are described below in ((1) to (6)) [27]. These reactions are dependent on the reduction potential of the metal involved and pH:

(1) $${NO}_{3(aq)}^{-}+{Zn}_{\left(s\right)}+H_{2}O_{\left(l\right)}\to {NO}_{2(aq)}^{-}+{Zn}_{\left(aq\right)}^{2+}+{2\;OH}_{\left(aq\right)}^{-}\;\Delta E=0.7718\;V$$

(2) $${2\;NO}_{3(aq)}^{-}+3{\;Zn}_{\left(s\right)}+8{\;H}_{\left(aq\right)}^{+}\to 2\;NO+{3\;Zn}_{\left(aq\right)}^{2+}+4{\;H}_{2}O_{\left(l\right)}\;\Delta E=1.7188\;V$$

(3) $${NO}_{3(aq)}^{-}+{Zn}_{\left(s\right)}+3{\;H}_{\left(aq\right)}^{+}\to H{NO}_{2(aq)}+{Zn}_{\left(aq\right)}^{2+}+H_{2}O_{\left(l\right)}\;\Delta E=1.6958\;V$$

(4) $${2\;NO}_{3(aq)}^{-}+{Zn}_{\left(s\right)}+{4\;H}_{\left(aq\right)}^{+}\to N_{2}O_{4}+{Zn}_{\left(aq\right)}^{2+}+2{\;H}_{2}O_{\left(l\right)}\;\Delta E=1.5648\;V$$

(5) $${2\;NO}_{3(aq)}^{-}+{Zn}_{\left(s\right)}+2{\;H}_{2}O_{\left(l\right)}\to N_{2}O_{4}+{Zn}_{\left(aq\right)}^{2+}+{4\;OH}_{\left(aq\right)}^{-}\;\Delta E=-0.088\;V$$

In the case of zinc, the reduction potential is given by [27]:

(6) $${Zn}_{\left(aq\right)}^{2+}+2\;e\to {Zn}_{\left(s\right)}E^{0}=-0.7618\;V$$

Species whose reduction potential is higher than −0.7618 V will be reduced by Zn^0^ [28]. The reduction reaction (1) will occur to a lesser extent compared with reactions (2)–(4), while reaction (5) is not spontaneous. Reactions (2)–(4) need higher levels of H^+^ than reaction (1), which means that reaction (1) occurs to a greater extent by controlling the pH. For this, a buffer solution with adequate buffering capacity can be used. This is important once, in an aqueous medium with a pH close to 6.00, the NO, $H{NO}_{2}$, and $N_{2}O_{4}$ species are less stable at room temperature than the ${NO}_{2(aq)}^{-}$. In addition, the method becomes more reproducible by ensuring the formation of a larger amount of the ${NO}_{2(aq)}^{-}$.

Nitrite can also be reduced by Zn^0^. Therefore, nitrite interferes in the reduction reaction of nitrate to nitrite according to the reactions (7) to (12) [27]:

(7) $$2\;H{NO}_{2}+{Zn}_{\left(s\right)}+2\;H_{\left(aq\right)}^{+}\to 2\;NO+{Zn}_{\left(aq\right)}^{2+}+{2\;H}_{2}O_{\left(l\right)}\;\Delta E=1.7448\;V$$

(8) $$2\;H{NO}_{2}+{2\;Zn}_{\left(s\right)}+{4\;H}_{\left(aq\right)}^{+}\to H_{2}N_{2}O_{2}+{2\;Zn}_{\left(aq\right)}^{2+}{+\;2\;H}_{2}O_{\left(l\right)}\;\Delta E=1.6218\;V$$

(9) $$2\;H{NO}_{2}+{2\;Zn}_{\left(s\right)}+{4\;H}_{\left(aq\right)}^{+}\to N_{2}O+{2\;Zn}_{\left(aq\right)}^{2+}+\;{2\;H}_{2}O_{\left(l\right)}\;\Delta E=2.0588\;V$$

(10) $${2\;NO}_{2(aq)}^{-}+{Zn}_{\left(s\right)}+\;{2\;H}_{2}O_{\left(l\right)}\to 2\;NO+{2\;OH}_{\left(aq\right)}^{-}\;\Delta E=0.3018\;V$$

(11) $${2\;NO}_{2(aq)}^{-}+{2\;Zn}_{\left(s\right)}+\;{2\;H}_{2}O_{\left(l\right)}\to N_{2}O_{2}^{-}+{{2\;Zn}_{\left(aq\right)}^{2+}+4\;OH}_{\left(aq\right)}^{-}\;\Delta E=0.5818\;V$$

(12) $${2\;NO}_{2(aq)}^{-}+{2\;Zn}_{\left(s\right)}+{3\;H}_{2}O_{\left(l\right)}\to N_{2}O+{{2\;Zn}_{\left(aq\right)}^{2+}+6\;OH}_{\left(aq\right)}^{-}\;\Delta E=0.9118\;V$$

Reactions (7) to (12) are spontaneous and occur in the presence of Zn^0^ [28]. Once reactions (7) to (9) need an acid medium, buffering at a pH = 6.00 ensures that these reactions occur to a lesser extent when compared to reaction (1). However, at pH = 6.00, reactions (10) to (12) may occur to a lesser extent compared with reaction (1). Therefore, the reduction reaction of nitrate to nitrite does not stop after the formation of nitrite, as nitrite can still be reduced according to reactions (10) to (12).

As explained above, nitrite must be eliminated from the sample, as it is not possible to guarantee that it will not be reduced by Zn^0^, thus subtracting it from the amount of nitrite generated by the nitrate reduction reaction. An efficient way to eliminate nitrite is the use of sulfamic acid, according to the reaction below:

(13) $$H_{3}{NSO}_{3(aq)}+{2NO}_{2(aq)}^{-}\to N_{2}+{SO}_{4(aq)}^{2-}+H_{2}O_{\left(l\right)}+H_{\left(aq\right)}^{+}$$

**Figure 2:**
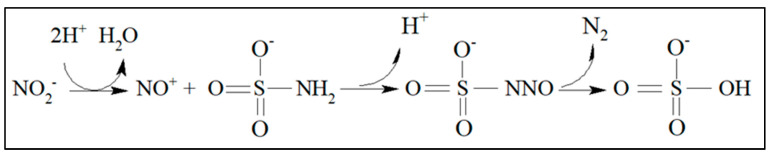
Sulfamic acid mechanism reaction for nitrite elimination from water samples.

According to its mechanism (shown in Figure 2), reaction (13) will only happen if the medium is acidic since the generation of nitronium ions only occurs in the presence of H^+^ [29]. Therefore, the reduction reaction of nitrate to nitrite can be carried out in the presence of sulfamic acid at pH = 6.00 without reaction (13) taking place. As sulfamic acid is a strong acid, the pH of the sample ranges from 5.5 to 6.0 after removing the nitrite once the buffer solution used in the procedure below (Section 3.2) was not completely consumed.

**Figure 3:**
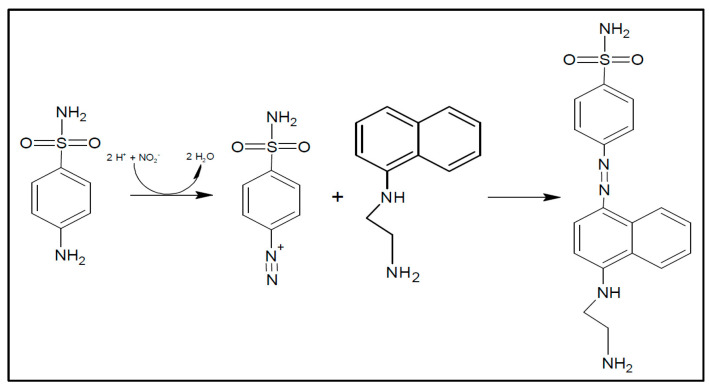
The Griess reaction for nitrite quantification.

It is necessary to add excess sulfamic acid for the complete elimination of nitrite, as per reaction (13). Assuming that the pH is between 5 and 8, 1 mL of a 6.0 g L^−1^ sulfamic acid solution must be added to 10 mL of a solution containing 1 mg L^−1^ of nitrite for complete elimination. For real samples, it is necessary to measure their pH and verify that they do not contain high alkalinity, as carbonates, bicarbonates, and alkaline compounds can consume sulfamic acid. An effective way to ensure the complete elimination of nitrite in real samples is to run a test using the Griess reaction (Figure 3) after the nitrite elimination until the analytical signal remains similar to blanks.

### 3.2. Influence of Buffer Solution Concentration and pH

As mentioned above, the concentration of H^+^ is a critical factor in the effectiveness of nitrate-to-nitrite reduction. The use of a buffer solution ensures the control of pH and, consequently, a sufficient concentration of H^+^. In this sense, preliminary tests were carried out using different types of buffer solutions at a pH similar to the pK_a_ of the respective conjugate acid. The efficiency of the nitrate-to-nitrite reduction reaction was only satisfactory when acetate–acetic acid or citrate–citric acid buffers were used. The acetic acid–acetate buffer was chosen because it requires less dissolution time, can be prepared at high concentrations without turbidity, and is cheaper.

**Figure 4:**
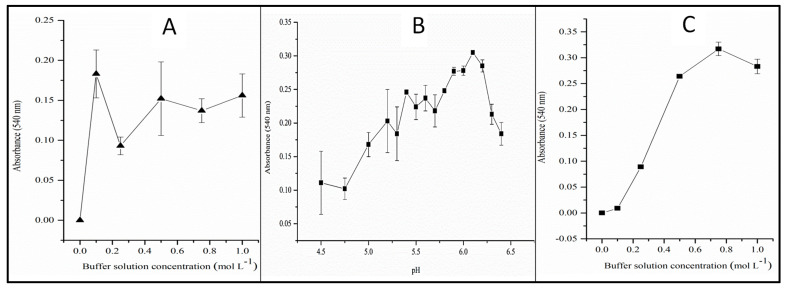
Influence of the concentration of the buffer solution and pH on the efficiency of the reaction to reduce nitrate to nitrite by metallic zinc. (**A**) Buffer solutions were prepared by varying only the initial concentration of acid and conjugate base (ratio 1:1), pH ~4.75. (**B**) pH variation on the efficiency of nitrate-to-nitrite reduction was performed using a 0.75 mol L^−1^ buffer solution. (**C**) Buffer solutions were prepared by varying the initial concentration of acid and conjugate base (ratio 1:1) at pH 6.00 for all buffer solutions.

The tests described above also showed that the maximum efficiency of the nitrate-to-nitrite reduction reaction depends on the buffer concentration and on the final pH (Figure 4A–C). In addition, preliminary tests indicated that the conditions and time of agitation and Zn^0^ mass of an adequate granulometry increase the reduction of nitrate to nitrite.

Acetic acid has a pK_a_ of 4.75 [30]. Although the pH = 6.00 of the reaction medium for the reduction of nitrate to nitrite was higher than the pK_a_ of acetic acid, the buffering capacity of the buffer solution was maximized by increasing the presence of the weak acid (CH_3_COOH, Figure 4). Furthermore, at pH = 6.00, the buffering capacity is approximately 0.02 [30,31]. At pH = 4.75, the buffering capacity is 0.05 [30,31]. This explains why relatively high concentrations (see Figure 4B,C), close to 1.0 mol L^−1^, are necessary for the nitrate-to-nitrite reduction reaction to have maximum efficiency at pH = 6.10. It is worth mentioning that the pH of a buffer solution varies as a function of the ratio between the concentrations of the weak acid and its conjugate base [30]. Therefore, the initial concentrations of the species in the buffer solution are different after adjusting the pH of this solution. In this work, the ratio between the initial concentrations of the weak acid and its conjugate base when preparing the buffer solution was ~1. Therefore, the expression “concentration of the buffer solution” refers to the initial concentrations of the weak acid and the conjugate base used for the buffer solution.

### 3.3. Univariate Studies of the Reduction Reaction of Nitrate to Nitrite

The univariate optimization consisted of studying the influence of the variables pH, buffer solution concentration, Zn^0^ mass, and agitation time. For this, standard nitrate solutions of 1 mg L^−1^ were used and proceeded as described in Figure 1. The efficiency of the nitrate reduction reaction to nitrite was evaluated by absorbance, according to the Griess reaction in Figure 3. The amount of colored diazo is proportional to the amount of nitrite generated in the nitrate reduction reaction.

Figure 4 shows the results of univariate optimizations to test the influence of the buffer solution concentration and pH on the efficiency of the nitrate-to-nitrite reduction by Zn^0^. To obtain the data in Figure 4A, buffer solutions were prepared by varying the initial concentrations of acetic acid and acetate (ratio 1:1 and pH ~4.75 for all solutions). The data in Figure 4A show that the absorbance varied practically within the margins of error. Based on this, the pH was evaluated (Figure 4B) using the initial concentration of 0.75 mol L^−1^. Figure 4B shows that pH also influences the efficiency of the reaction. Absorbance increases with pH to a maximum value close to 6.10. Below this value, the concentration of H^+^ ions favored the undesirable reactions (10) to (12). At pH values above 6.10, the concentration of H^+^ is insufficient for reaction (1) to achieve maximum efficiency. In addition, the error bars are smaller for values between 5.80 and 6.10. This demonstrates that the pH has a strong influence on the efficiency and reproducibility of the nitrate-to-nitrite reduction, which worsens the precision. Finally, we tested the same buffer concentrations (0.0 to 1.0 mol L^−1^) at pH 6. The results are in Figure 4C. The efficiency of the reaction varied with the initial concentration of the weak acid and its conjugate base, reaching a maximum value of 0.80 mol L^−1^. Therefore, based on the data in Figure 4, the values of the initial concentration of weak acid and its conjugate base (ratio 1:1) and pH that provide maximum efficiency in the reduction of nitrate to nitrite by Zn^0^ are, respectively, 0.80 mol L^−1^ and 6.00.

**Figure 5:**
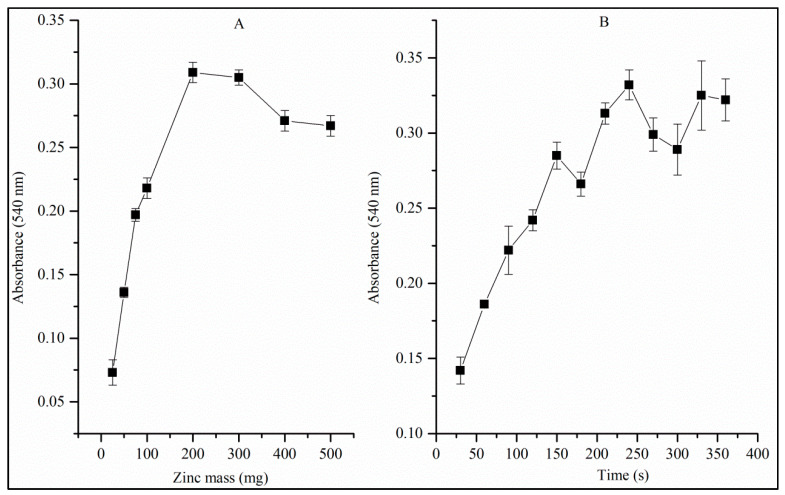
Influence of mass of Zn^0^ on the efficiency of the nitrate-to-nitrite reduction reaction with agitation for 4 min (**A**) and influence of time on the efficiency of the nitrate-to-nitrite reduction reaction using metallic zinc with 20 MESH particle size (**B**).

Figure 5A shows absorbance as a function of zinc mass. Preliminary studies have shown that the reduction reaction of nitrate to nitrite by Zn^0^ does not occur efficiently if the Zn^0^ is present in the form of pellets, chips, small spheres or chopped wire. Zn^0^ powder, despite providing good efficiency in the reaction, has a very large surface area and, therefore, decomposes the diazo compound generated in a few seconds (Figure 2). To avoid this decomposition, a filtration step was proposed to eliminate Zn dust [16]. However, this filtration step increases the time and cost of analysis. In the present study, 20 MESH Zn^0^ particles were used without filtration, which is a time and cost problem for routine analysis. Using 20 MESH Zn^0^, no interference was observed in the stability of the diazo compound for at least one hour.

According to Figure 5A, there is a region approximately between 100 mg and 300 mg in which the reduction of nitrate to nitrite has maximum efficiency. Zinc mass above 300 mg favored the excessive reduction of the generated nitrite, according to reactions (10) to (12); moreover, Zn > 300 mg makes it difficult to measure the absorbance due to the reflection of light promoted by the large number of suspended Zn particles even after 10 min (time for the Griess reaction to occur). Therefore, 300 mg was chosen for further studies.

Figure 5B shows absorbance as a function of agitation time. Above 200 s, the efficiency of the reduction of nitrate to nitrite is at its maximum. Therefore, an agitation time of 240 s is necessary to guarantee maximum efficiency. We noticed that agitation accelerates the process of the microparticulate Zn^0^, releasing products and exposing a fresh Zn^0^ surface to continue the reaction and obtain better results even at low nitrate concentrations. 

Based on what was exposed above in this section, it can be concluded that there is a range of buffer solution concentration, pH, mass of Zn^0^, and agitation time in which the efficiency of the reduction reaction from nitrate to nitrite is at its maximum. The conditions chosen for the method were buffer solution concentration = 0.75 mol L^−1^, pH = 6.10, mas of Zn^0^ = 300 mg, and agitation time = 4 min. 

## 4. Method Validation

### 4.1. Selectivity

**Figure 6:**
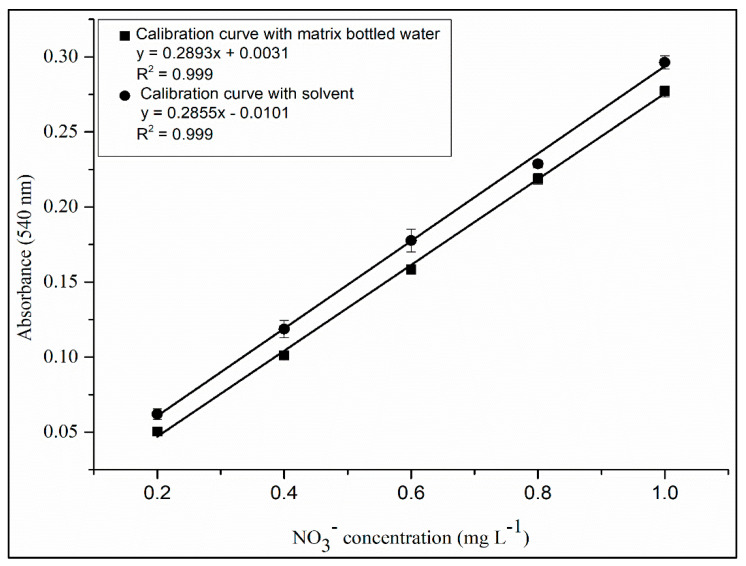
Nitrate calibration curves for the matrix and for the solvent obtained in the study of the matrix effect.

The *t* test and *F* test presented values of 0.53 and 1.65, respectively, which are smaller than the tabulated values. In addition, Figure 6 shows the lines of the calibration curves with and without matrix matching. Therefore, the curves have statistically equal slopes, confirming the selectivity of the method [22,25].

### 4.2. Working Range

**Figure 7:**
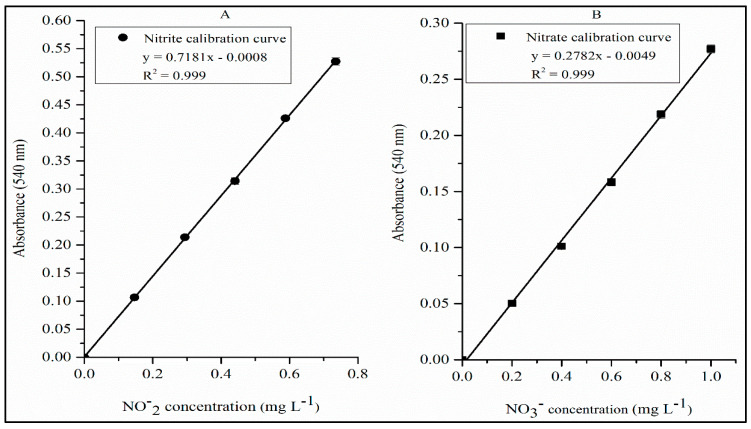
Calibration curve for nitrite (**A**) and nitrate (**B**) quantification in bottled water samples.

The first concentration of the analytical curve for nitrate is close to the LoQ (Figure 7). Samples with an analytical signal below the lowest concentration of the analytical curve were considered non-quantifiable. Once the limit for nitrate in drinking water is 10 mg L^−1^ [7,8], dilutions of 10× for samples in the limit will be in the range. Samples above this limit require higher dilutions, not compromising the uncertainty of the method. For calibrations using concentrations above 1 mg L^−1^ of nitrate, some parameters tested in the proposed method are not sufficient to guarantee the maximum efficiency of nitrate-to-nitrite reduction and detection. Moreover, for concentrations of >1 mg L^−1^, it would be necessary to increase the mass of Zn^0^, the concentration of buffer solution, and the stirring time. For example, a Zn^0^ mass of >350 mg interferes with spectrophotometric measurements. Therefore, a working range between 0.20 mg L^−1^ and 1 mg L^−1^ was the best range for the proposed method.

### 4.3. Linearity

The chi-square test for White’s test was 4.15. This value is lower than the tabulated value (5.99) [32]. This guarantees that there are only homoscedastic errors [32]. Based on this, the ordinary least squares method could be used to obtain the best straight line of the linear regression [20,21,22]. Figure 7A,B shows the analytical curves obtained for nitrite and nitrate analysis in samples. The R^2^ values of 0.999 for the curves were very close to 1, which indicates excellent linearity [19,20,21,24].

**Figure 8:**
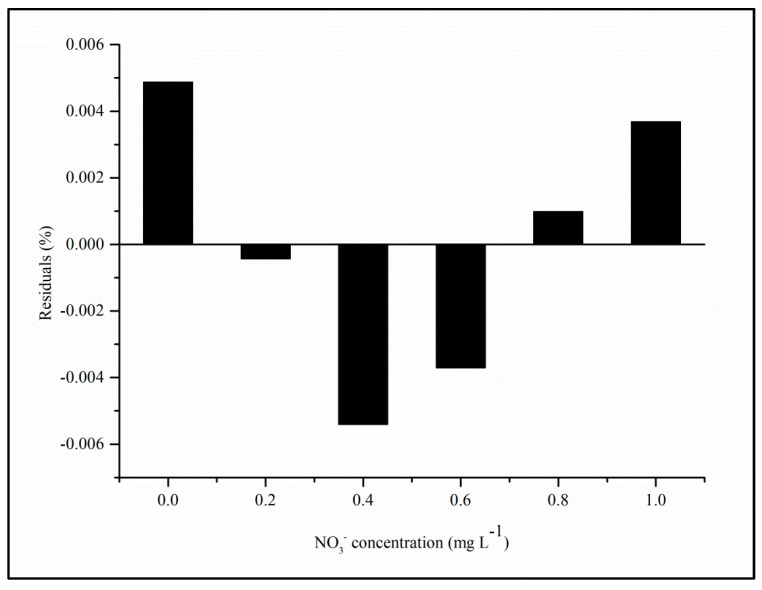
Regression plot of residuals from the calibration curve for nitrate quantification (refer to Figure 7B).

In addition to the two parameters mentioned above, according to Figure 8, the analyses of the residuals of the regression of the analytical curve (from Figure 7B) show that they were randomly distributed, which also confirms the linearity of the proposed method [20].

### 4.4. Limit of Detection and Limit of Quantification 

The LoD and LoQ results were, respectively, 0.024 mg L^−1^ and 0.08 mg L^−1^. These values are lower than those of similar methods reported in the literature. Merino [16] and Murray et al. [17] found LoDs of 5 mg L^−1^ and 0.5 mg L^−1^, respectively. In addition, the LoQ of the proposed method perfectly meets the maximum limit of nitrate allowed in water by Brazilian legislation by two orders of magnitude. This is extremely important, as this maximum limit can be decreased by authorities (from future toxicological studies).

### 4.5. Bias

Table 1 shows the results of the recovery tests for the three studied concentrations. For methods that quantify chemical species at 1 mg L^−1^ to be considered biased, the recovery must be in the range of 80% to 110% [19,24]. Therefore, the results in Table 1 prove that the bias in the proposed method is within the recovery range recommended by the literature.

In addition to recovery tests, bias was determined by comparing the proposed method with the official method (IC). The results obtained for the *F* test and the paired t were lower than those tabulated. Therefore, there is no significant bias in the proposed method in relation to the results produced by the current IC method.

### 4.6. Uncertainty

Relative uncertainties, with the exception of samples B and L, were ≤10.32%. This is in line with official method validation protocols, which recommend a 20% to 30% tolerance on relative uncertainties [23]. According to Table 2, these uncertainties decrease with increasing nitrate concentration and are greater than 30% for concentrations close to the LoQ of the method. Therefore, the method has its precision and accuracy compromised for nitrate concentrations close to the LoQ [23]. Based on this, one can also understand the importance of making the analytical curve with the first standard solution with a concentration slightly above the LoQ, ensuring precision and accuracy.

### 4.7. Precision

Precision was evaluated by studying repeatability and intermediary precision. The results for the relative standard deviations (RSDs) are in Table 2. The values were below 5%, which allows us to conclude that the precision at the repeatability level of the proposed method is in agreement with the recommended one [21,22,24].

Intermediate precision was evaluated by performing samples analyses in different days (2 different days of analysis). In addition, analyses were also performed on these samples using reagents from different brands and lots and different spectrophotometers, with three replicates per sample and six measurements on the equipment. The results for the *F* test were lower than the tabulated. Therefore, there is no significant difference between the variances. Therefore, it can be concluded that the proposed method presented the necessary intermediate precision as recommended in the literature [19,21,22,24].

### 4.8. Quantification of Nitrate in Samples

Table 2 shows the results of nitrate and nitrite quantification in the 20 bottled water samples. Only one sample showed a result for nitrite but with a concentration below the maximum limit established by national legislation. Regarding nitrate, no sample showed a higher value than the maximum allowed by national legislation. Therefore, all samples comply with the maximum concentrations of nitrate and nitrite allowed in mineral water. The results in Table 2 also show a variation in the nitrate concentration in the samples from a concentration close to the LoQ of the proposed method to a concentration above the last standard of the analytical curve in Figure 7. Thus, the proposed method is able to quantify concentrations ranging from 0.2 mg L^−1^ to concentrations two orders of magnitude above this value.

## 5. Conclusions

The proposed method is valid for quantifying nitrate in bottled mineral water using visible spectrophotometry, and the results of the validation study show that the method fulfills the internationally accepted fitness for purpose criteria for precision and trueness. In addition, the LoD and LoQ were lower compared with other spectrophotometric methods. The use of Zn^0^ as a reducing agent replaces the highly toxic metallic cadmium and contributes to a more ecological environment and a safer procedure. In addition, the proposed procedure minimizes the toxicity of discards of reagents used for nitrate determinations routinely performed in laboratories around the world. Acetate–acetic acid buffered reaction medium is a less toxic reagent (for the analyst and the environment) compared with ammonium buffer. Moreover, the acetate–acetic acid buffer can be easily prepared and transported as stable stock solutions. The cost advantage (that is, a relatively less expensive) and easy-to-use procedure can be present at routine laboratories and is easy to use in portable commercial low-weight colorimeters capable of measuring at 540 nm, performing suitable measurements inside and outside labs.

## Figures and Tables

**Table 1:** Results of the recovery tests for the accuracy of three concentrations.

Level	Mean Concentration (mg L^−1^)	Recovery (%)	RSD (%)
Low (0.3 mg L^−1^)	0.27	86.35	4.15
Medium (0.6 mg L^−1^)	0.52	85.9	2.75
High (1.0 mg L^−1^)	0.85	84.61	1.1

**Table 2:** Nitrate and nitrite concentrations in bottled water samples. The analyses were carried out according to the proposed method.

Sample	Nitrate Mean Concentration (mg L^−1^)	Uncertainty (mg L^−1^)	Relative Uncertainty (%)	RSD (%)
A	3.38	0.08	2.39	1.06
B	0.12	0.08	71.58	3.95
C	1.73	0.15	8.56	1.91
D	<LoQ	-	-	-
E	2.01	0.15	7.31	1.80
F	0.79	0.08	10.32	0.88
G	19.37	0.08	0.42	0.31
H	2.36	0.08	3.39	0.44
I	28.20	0.08	0.28	1.69
J	4.79	0.08	1.67	1.15
K	4.01	0.08	2	1.03
L *	0.13	0.08	66.18	3.66
M	2.87	0.08	2.80	0.48
N	5.04	0.08	1.59	0.82
O	2.94	0.08	2.73	0.93
P	5.74	0.08	1.40	2.01
Q	0.89	0.08	9.24	1.58
R	12.21	0.08	0.66	0.59
S	0.91	0.08	9.08	1.17
T	11.06	0.08	0.72	1.95

* Nitrite (mg L^−1^): all samples presented values < LoQ, except the sample L (0.10 mg L^−1^).

## Data Availability

Data are contained within the article.
